# Dynamic susceptibility-contrast magnetic resonance imaging with contrast agent leakage correction aids in predicting grade in pediatric brain tumours: a multicenter study

**DOI:** 10.1007/s00247-021-05266-7

**Published:** 2022-03-15

**Authors:** Stephanie B. Withey, Lesley MacPherson, Adam Oates, Stephen Powell, Jan Novak, Laurence Abernethy, Barry Pizer, Richard Grundy, Paul S. Morgan, Simon Bailey, Dipayan Mitra, Theodoros N. Arvanitis, Dorothee P. Auer, Shivaram Avula, Andrew C. Peet

**Affiliations:** 1 RRPPS, University Hospitals Birmingham NHS Foundation Trust, Birmingham, UK; 2 Oncology, Birmingham Women’s and Children’s NHS Foundation Trust, Birmingham, UK; 3 Institute of Cancer and Genomic Sciences, University of Birmingham, Birmingham, UK; 4 Radiology, Birmingham Women’s and Children’s NHS Foundation Trust, Birmingham, UK; 5 Department of Psychology, Aston Brain Centre, School of Life and Health Sciences, Aston University, Birmingham, UK; 6 Radiology, Alder Hey Children’s NHS Foundation Trust, Liverpool, UK; 7 Oncology, Alder Hey Children’s NHS Foundation Trust, Liverpool, UK; 8 The Children’s Brain Tumour Research Centre, University of Nottingham, Nottingham, UK; 9 Medical Physics, Nottingham University Hospitals, Nottingham, UK; 10 Division of Clinical Neuroscience, School of Medicine, University of Nottingham, Nottingham, UK; 11 Sir James Spence Institute of Child Health, Royal Victoria Infirmary, Newcastle upon Tyne, UK; 12 Neuroradiology, Royal Victoria Infirmary, Newcastle upon Tyne, UK; 13 Institute of Digital Healthcare, WMG, University of Warwick, Coventry, UK; 14 Neuroradiology, Nottingham University Hospitals Trust, Nottingham, UK; 15 NIHR Nottingham Biomedical Research Centre, Nottingham, UK; 16 Children’s Brain Tumour Research Team, 4th Floor Institute of Child Health, Birmingham Women’s and Children’s Hospital NHS Foundation Trust, Steelhouse Lane, Birmingham, B4 6NH UK

**Keywords:** Blood volume, Brain, Children, Dynamic susceptibility-contrast magnetic resonance imaging, Leakage correction, Magnetic resonance imaging, Multicenter, Perfusion, Tumor

## Abstract

**Background:**

Relative cerebral blood volume (rCBV) measured using dynamic susceptibility-contrast MRI can differentiate between low- and high-grade pediatric brain tumors. Multicenter studies are required for translation into clinical practice.

**Objective:**

We compared leakage-corrected dynamic susceptibility-contrast MRI perfusion parameters acquired at multiple centers in low- and high-grade pediatric brain tumors.

**Materials and methods:**

Eighty-five pediatric patients underwent pre-treatment dynamic susceptibility-contrast MRI scans at four centers. MRI protocols were variable. We analyzed data using the Boxerman leakage-correction method producing pixel-by-pixel estimates of leakage-uncorrected (rCBV_uncorr_) and corrected (rCBV_corr_) relative cerebral blood volume, and the leakage parameter, K_2_. Histological diagnoses were obtained. Tumors were classified by high-grade tumor. We compared whole-tumor median perfusion parameters between low- and high-grade tumors and across tumor types.

**Results:**

Forty tumors were classified as low grade, 45 as high grade. Mean whole-tumor median rCBV_uncorr_ was higher in high-grade tumors than low-grade tumors (mean ± standard deviation [SD] = 2.37±2.61 vs. –0.14±5.55; *P*<0.01). Average median rCBV increased following leakage correction (2.54±1.63 vs. 1.68±1.36; *P*=0.010), remaining higher in high-grade tumors than low grade-tumors. Low-grade tumors, particularly pilocytic astrocytomas, showed T1-dominant leakage effects; high-grade tumors showed T2*-dominance (mean K_2_=0.017±0.049 vs. 0.002±0.017). Parameters varied with tumor type but not center. Median rCBV_uncorr_ was higher (mean = 1.49 vs. 0.49; *P*=0.015) and K_2_ lower (mean = 0.005 vs. 0.016; *P*=0.013) in children who received a pre-bolus of contrast agent compared to those who did not. Leakage correction removed the difference.

**Conclusion:**

Dynamic susceptibility-contrast MRI acquired at multiple centers helped distinguish between children’s brain tumors. Relative cerebral blood volume was significantly higher in high-grade compared to low-grade tumors and differed among common tumor types. Vessel leakage correction is required to provide accurate rCBV, particularly in low-grade enhancing tumors.

## Introduction

Dynamic susceptibility-contrast MRI is a technique to measure perfusion in the brain. It involves the injection of a contrast agent during rapid MRI scanning, resulting in T2- or T2*-weighted signal changes as the bolus of contrast agent passes through the intravascular space. Relative cerebral blood volume (rCBV) can be calculated by integrating the contrast agent concentration–time curve and is usually reported in tumors as normalized to normal white matter [1, 2]. Relative cerebral blood volume has been shown to be useful for grading pediatric brain tumors [1, 2], monitoring treatment response [3], differentiating recurrent/residual tumor from treatment effect and providing markers of long-term prognosis [4]. Studies are usually performed at a single center, so there is a need to boost study numbers via multicenter studies and demonstrate that the technique is reproducible across sites and scanners to inform its use in clinical practice.

Conversely to adult brain tumors, many low-grade pediatric brain tumors display significant contrast enhancement on T1-weighted images [5]. Calculation of rCBV assumes that the blood–brain barrier remains intact. This is often not the case in brain tumors. Contrast agent leakage from the intravascular to the extravascular extracellular space results in an increase in MR signal from T1 shortening and underestimation of rCBV. Conversely, T2 and T2* effects arise when there are changes in susceptibility differences between tissue compartments, reducing the MR signal so that it does not recover to baseline. This results in overestimation of rCBV. T1 effects can be reduced by administering a contrast agent pre-bolus [6, 7], by careful choice of pulse sequence parameters [6] or by post-processing methods [8–10]. A lack of agreement on the optimum acquisition technique leads to variations in protocols across centers and challenges for multicenter studies.

Following identification of a brain tumor on MRI, most children undergo biopsy or surgical resection. Some brain tumor types, such as diffuse intrinsic pontine gliomas [11], cannot be biopsied or undergo surgery because of their midline position. In addition, histopathological diagnosis has its limitations — results can be inconclusive or require central review, leading to increased waiting times for a diagnosis [12]. Treatment decisions are made based on tumor type, grade, molecular subtype, spread and age and fitness of the patients.

Advanced MRI techniques, such as magnetic resonance (MR) spectroscopy [12] and diffusion-weighted imaging [13], have been shown to increase accuracy in diagnosis compared to conventional MRI, resulting in increased confidence of radiological reporting. Early noninvasive diagnosis using advanced MRI can provide additional information and confidence in diagnoses compared to standard MRI alone, and is particularly useful in cases where biopsy/surgery is not an option or histological results are delayed. It can also inform the optimal biopsy site and allow for timely family discussions and organization of treatment.

Single-center dynamic susceptibility-contrast MRI studies [1, 2, 14, 15] have shown significantly higher rCBV associated with high-grade pediatric brain tumors, but patient numbers are small. In addition, differences in MR scanners and protocols mean that parameters are not always comparable among centers. There is a need for multicenter pediatric studies to investigate the effect that differences in scanners and protocols have on perfusion parameters in order to develop robust biomarkers that can be used clinically to help with noninvasive tumor grading. The aims of this study were to (1) compare dynamic susceptibility-contrast MRI parameters in newly diagnosed pediatric low- versus high-grade tumors, with and without leakage correction; and (2) compare data acquired at multiple centers using varying dynamic susceptibility-contrast MRI protocols. We hypothesized that rCBV acquired from multicenter dynamic susceptibility-contrast MRI studies of children’s brain tumors differs between high- and low-grade tumors and that leakage correction is important in determining rCBV.

## Materials and methods

This study was approved by the East Midlands–Derby research ethics committee (NRES REC ref.: 04/MRE04/41) and was performed in accordance with the ethical standards as laid down in the 1964 Declaration of Helsinki (and as revised in 1983). Informed parental consent was obtained from all subjects. Suitable patients were those undergoing dynamic susceptibility-contrast MRI scans with a primary brain tumor where histological data — tumor type and grade — were subsequently obtained. We anonymized the dynamic susceptibility-contrast and clinical MRI scans and uploaded them to the Children’s Cancer and Leukaemia Group [16] Functional Imaging Database.

### Magnetic resonance imaging protocols

We acquired data from four centers that used six different MRI scanners. The protocols on each scanner are summarized in Table 1. Although dynamic susceptibility-contrast and clinical protocols were recommended by the European Society for Paediatric Oncology [17], centers chose and set up their own scanner-dependent protocols. Most centers used a gradient echo echoplanar imaging sequence with variable time to repetition (TR). Flip angles varied, with a low flip angle chosen to minimize T1-leakage effects on the MR signal. Scanning continued for a minimum of 70 s. While most centers covered the whole brain, this was not universal. Spatial resolution was variable. One center predominantly used the sensitivity-encoded Philips sPRESTO (principles of echo-shifting with a train of observations) sequence [18].

**Table 1:** Summary of seven dynamic susceptibility-contrast MRI protocols run across six scanners at four centers in this multicenter study

Center	1		2	3	4		
Scanner type	Siemens Avanto	Philips Achieva	Siemens Verio	Philips Achieva	Philips Achieva	Philips Achieva	Philips Achieva
Field strength	1.5 T	3 T	3 T	3 T	1.5 T	3 T	3 T
Head coil	12-element head	32-channel	32-channel	SENSE head-8	SENSE-NV-16	SENSE head-8	SENSE head-8
Sequence	GE-EPI	GE-EPI	GE-EPI	GE-EPI	sPRESTO	sPRESTO	GE-EPI
TR (ms)	1,490–1,643	1,830–1,865	1,570	1,666–2,343	16.7–17.2	15.5–16.0	582–1,866
TE (ms)	40	40	29	40	24.7–25.2	23.5–24.0	18.4–40.0
Flip angle (^o^)	20	20	45	75	7	7	20–40
Slice thickness (mm)	5.0	3.5	3.5	4.0	3.5	3.5	3.5–7.0
No. slices	19–21	30	16	25–35	30–36	30–34	30
No. dynamics	60	60	60	40	60	60	40–60
Field-of-view (mm)	230×230	240×240	220×220	224×224	220×220	230×230	240×240
Matrix	96×96	96×96	64×64	128×128	64–80×64–80	128×128	96×96
SENSE?	Y	Y	N	Y	Y	Y	Y
Temporal resolution (s)	1.5–1.6	1.8–1.9	1.6	1.7–2.3	1.3–1.6	1.2–1.4	0.6–1.9
Total scan time (s)	90–99	110–112	94	67–94	77–94	71–83	70–118
Pre-bolus	Y	Y	N	Y	Y (*n*=12), N (*n*=15), NA (*n*=9)		
Pre-bolus dose	Half	Half	–	1 mL	Range = 0.3–2.0 mL (10% of dose)		
Injection rate (mL/s)	3	3	6	3	3	3	3
Contrast agent	Dotarem	Dotarem	Dotarem	Dotarem	Magnevist (*n*=20), Gadovist (*n*=10), NA (*n*=6)		
No. patients	8	3	12	26	15	16	5

*GE-EPI* gradient echo echoplanar imaging, *n* number, *N* no, *NA* not available, *No.* number, *SENSE* sensitivity encoding, *sPRESTO* sensitivity-encoded Philips principles of echo-shifting with a train of observations, *T* tesla, *TE* time to echo, *TR* time to repetition, *Y* yes

Gadolinium-containing contrast agent was administered via power injector through a cannula inserted into a suitable vein. Contrast agent brand, dose and injection rate are summarized (Table 1). Centers 1 and 3 consistently gave a pre-bolus of 50% of total dose and 1 mL, respectively, administering the total dose of contrast in two stages — a pre-bolus for minimization of T1 effects followed by a second dose during the dynamic susceptibility-contrast data acquisition. An injection rate of 3 mL/s was recommended to minimize dispersion of the contrast agent bolus [17] and was used by all sites except one, which used a higher injection rate of 6 mL/s. Each injection was followed by up to 10 mL of saline. Center 2 did not employ a pre-bolus; at center 4, a pre-bolus of 10% of the total dose was administered to some children, depending on scanner, child’s age and whether the child was scanned under general anesthetic. Injection details were not recorded for nine patients.

### Histology

Children underwent surgical resection or biopsy following MRI. Histological diagnoses were obtained locally. Tumors were classified and graded according to World Health Organization guidance available at the time [19–21] and were subsequently classified as low-grade (I and II) or high-grade (III and IV).

### Data analysis

**Fig. 1:**
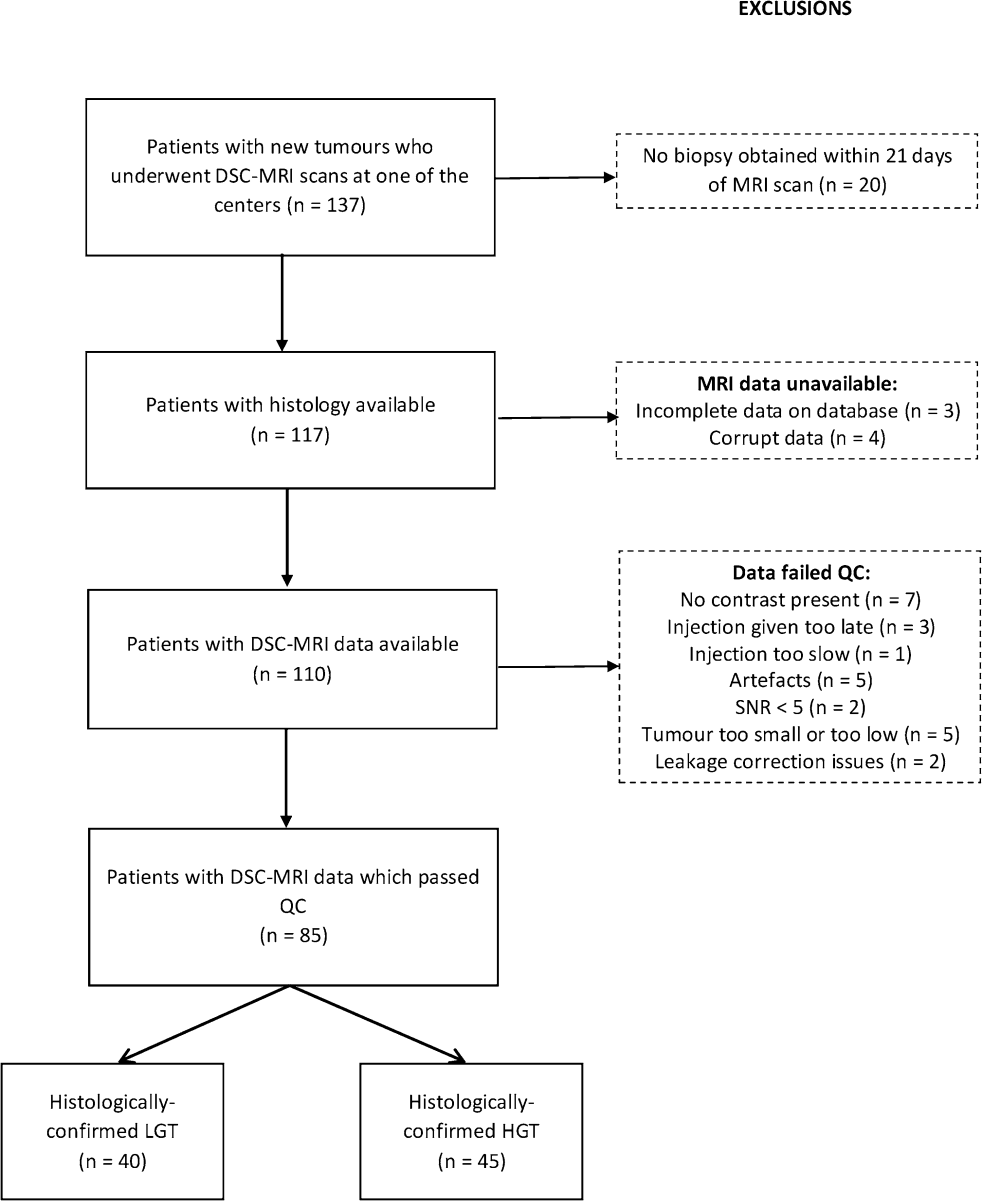
Flow diagram shows participants included in the study and reasons for exclusions. *DSC-MRI* dynamic susceptibility-contrast magnetic resonance imaging, *HGT* horizontal gene transfer, *LGT* lateral gene transfer*, QC* quality criteria, *SNR* signal-to-noise ratio

We checked the MRI data downloaded from the central database for quality and completeness. We rejected data if scans were missing from the database, the contrast agent injection was administered late or slow so that there was insufficient acquisition to view the bolus passage, there were problems loading the data into the analysis software, or there were significant image artifacts or difficulties applying leakage-correction (Fig. 1).

Dynamic susceptibility-contrast analysis was performed using in-house software written in the Python programming language (v. 2.7). Pixel averaging using a 3×3 Gaussian kernel was performed prior to extracting signal–time curves from the dynamic susceptibility-contrast time-course on a pixel-by-pixel basis. Signal–time curves were converted to change in T2* relaxation time, ΔR2^*^:

1 $$\Delta {R}_2\ast (t)=-\frac{1}{TE}\mathit{\ln}\left(\frac{S(t)}{S(0)}\right)$$

*S(t)* and *S(0)* are the signal intensities at time *t* and baseline, respectively; *TE* is the time-to-echo of the dynamic susceptibility-contrast sequence. S(0) was calculated by averaging the signal from the first 6 time points. Pixel-by-pixel uncorrected cerebral blood volume (CBV_uncorr_) values were calculated by integrating over the DR2*-time curves. Leakage-corrected DR2_,corr_*-time curves were calculated using the Boxerman method, which also estimates the leakage-correction parameter, K_2_ [8–10]. This model aims to correct for both T1 and T2* effects arising from contrast agent extravasation.

2 $$\Delta {R}_{2, corr}\ast (t)=\Delta {\overset{\sim }{R}}_2^{\ast }(t)+{K}_2{\int}_0^t\Delta {\overline{R}}_2^{\ast}\left({t}^{\prime}\right){dt}^{\prime }$$

$\Delta {\overset{\sim }{R}}_2^{\ast }(t)$ is the uncorrected DR2*, $\Delta {\overline{R}}_2^{\ast}\left({t}^{\prime}\right)$ is the DR2* obtained over the whole non-contrast-enhanced brain, therefore providing an estimate of DR2* without allowing for leakage. *K*_*2*_ is a term reflecting the effects of leakage on both T1 and T2* and is estimated by least-squares fitting the uncorrected DR2* to:

3 $$\Delta {\overset{\sim }{R}}_2(t)\equiv {K}_1\cdot \Delta {\overline{R}}_2^{\ast }(t)-{K}_2{\int}_0^t\Delta {\overline{R}}_2^{\ast}\left({t}^{\prime}\right) dt^{\prime }$$

Positive K_2_ indicates that T1 effects dominate the resulting signal–time curve, while negative K_2_ indicates T2*-dominant effect [9]. K_1_ is a constant of proportionality.

Pixels were included in $\Delta {\overline{R}}_2^{\ast }(t)$ as non-contrast-enhanced brain if they were not located in a ventricle, if the average signal of the last 10 time points was less than the average plus the standard deviation in the baseline and if the average baseline signal intensity was greater than background noise. A manually input threshold for each patient differentiated ventricle from surrounding brain tissue.

Corrected cerebral blood volume (CBV_corr_) was calculated by integrating over leakage-corrected DR2*–time curves, DR2_,corr_*(t). Maps of uncorrected and corrected cerebral blood volume and K_2_ were produced for each patient.

### Regions of interest

At center 1, a high-resolution T2-weighted turbo spin-echo scan with the same coverage as the dynamic susceptibility-contrast scan was acquired for defining regions of interest (TR/TE=4,000/100 ms, matrix = 144×144). At other centers T2-weighted and post-gadolinium T1-weighted clinical scans were downloaded from the central database for each patient depending on availability. Scans that did not have the same coverage as the dynamic susceptibility-contrast scan were reformatted and registered to the dynamic susceptibility-contrast images using an automatic affine transformation in the MERIT module in MeVisLab (v. 2.8.2; MeVis Medical Solutions AG, Bremen, Germany). After viewing the whole image set available for each case to clarify the tumor margins, whole-tumor regions of interest, excluding areas of cyst and vessels, were defined using MRIcro [22] by a Clinical Scientist with 15 years of experience (S.B.W.) trained by a Consultant Pediatric Radiologist with 17 years of experience (L.M.). If there was any doubt as to whether abnormal tissue was tumor, it was not included in the region of interest. Fourteen randomly selected patients had regions of interest redrawn by a Consultant Radiologist with 7 years of experience (A.O.) to assess reproducibility.

Regions of interest were also defined in supratentorial normal-appearing cerebral white matter. Mean white matter cerebral blood volume was calculated for each child and used to normalize CBV_uncorr_ and CBV_corr_ maps. We then applied tumor regions of interest as a mask to the white-matter-normalized rCBV maps. Whole-tumor volumes were calculated by multiplying the number of voxels in the whole-tumor region-of-interest by the voxel volume. We calculated median, standard deviation, minimum, maximum, skewness and kurtosis of normalized whole-tumor uncorrected and corrected rCBV and K_2_. We further divided the tumors listed in Table 2 into five groups by type: pilocytic astrocytomas, medulloblastomas, ependymomas, along with the less common high- and low-grade tumors grouped together as “other high-grade tumors” and “other low-grade tumors,” respectively. We calculated percentiles and produced histograms showing the distribution of whole-tumor parameters. Average rCBV_uncorr_, rCBV_corr_ and K_2_ histograms were calculated for each of the five tumor groups.

**Table 2:** Demographics for the 85 children scanned

Diagnosis	*n*	Gender M/F	Age, yrs. (mean±SD)	Grade(s)	Classification	Center				Mean±SD		
						1	2	3	4	Uncorrected median rCBV	Corrected median rCBV	Median K_2_
Pilocytic astrocytoma	27	13/14	8.3±4.9	1	Low-grade	2	4	8	13	0.13±2.23	1.53±1.24	0.018±0.034
Medulloblastoma	23	13/10	8.6±4.2	4	High-grade	4	4	5	10	1.80±1.53	2.28±1.17	0.004±0.016
Anaplastic astrocytoma	3	3/0	10.8±1.7	3	High-grade	–	2	–	1	1.15±0.38	1.26±0.48	0.002±0.004
Ependymoma^a^	9	3/6	4.8±4.4	2 and 3	High-grade	1	1	1	6	2.60±4.36	2.89±2.75	0.003±0.021
Central nervous system primitive neuroectodermal tumor	3	3/0	6.1±1.6	4	High-grade	–	–	3	–	4.09±1.63	3.49±1.67	−0.009±0.007
Oligodendroglioma	3	2/1	11.6±4.8	2	Low-grade	–	–	3	–	3.04±1.66	2.54±1.51	−0.022±0.041
Other high-grade tumors	7	4/3	7.7±6.3	3 and 4	High-grade	2	–	–	5	3.72±3.08	3.08±1.20	0.001±0.020
Other low-grade tumors	10	7/3	7.9±6.2	1 and 2	Low-grade	2	1	6	1	−1.81±10.58	1.81±1.64	0.026±0.077
Total	85	48/37	8.0±4.8			11	12	26	36	1.19±4.41	2.13±1.56	0.009±0.036

For analysis, the tumors listed here were further divided into five groups of the three most common types included in the study, with all remaining tumors grouped as “other low-grade tumors” and “other high-grade tumors.” Here, the “other high-grade tumor group” includes atypical teratoid rhabdoid tumor (*n*=1), glioblastoma (*n*=2), pineoblastoma (*n*=2) and choroid plexus carcinoma (*n*=2); the “other low-grade tumor group” includes dysembryoplastic neuroepithelial tumor (*n*=2), diffuse astrocytoma (*n*=2), choroid plexus papilloma (*n*=1), atypical choroid plexus papilloma (*n*=1), fibrillary astrocytoma (*n*=1), chordoma (*n*=1), pleomorphic xanthoastrocytoma (*n*=1) and craniopharyngioma (*n*=1)

*F* female, *K*_*2*_ leakage parameter, *M* male, *rCBV* relative cerebral blood volume, *SD* standard deviation, *yrs* years

^a^Grade II ependymomas were classified as high-grade tumors because clinically they are treated as malignant tumors [23]

### Statistical analysis

Statistical analyses were performed using SPSS (v. 2.5; IBM, Armonk, NY). A Shapiro-Wilk test was performed to test for normality. Spearman correlation was used to test for relationships between variables. We used a Wilcoxon signed-rank test to examine differences between parameters obtained from regions of interest defined by different operators. Kruskal-Wallis was performed to test for differences in parameters between the low- and high-grade tumor groups, among data acquired at different centers and among the five tumor groups. We also investigated sensitivity and specificity for distinguishing tumors as high- or low-grade using the median of each parameter as a cut-off.

## Results

The flow of participants through the study and reasons for exclusion are shown in Fig. 1. Table 1 summarizes the protocols of the four centers at which the 85 eligible children were scanned. Children were scanned between November 2005 and May 2017. Table 2 summarizes the demographics and diagnoses of eligible children. Data are shown for the most common tumor types, with the least common low- and high-grade tumors grouped together, respectively. Forty-five and 40 tumors were classified as high- and low-grade, respectively. Eleven, 12, 26 and 36 children were scanned at centers 1, 2, 3 and 4, respectively. The median time between the date of MRI scan and tissue being taken was 2 days (range 0–20 days). Forty-one children underwent complete macroscopic resection, 27 underwent incomplete resection and 17 underwent biopsy.

Wilcoxon signed-rank tests on whole-tumor median metrics — rCBV_uncorr_, rCBV_corr_ and K_2_ — obtained from regions of interest defined by two separate operators to assess reproducibility (*n*=14) showed no significant differences between the defined regions of interest (*P*>0.05 in all cases). There were no significant differences between the proportions of low- and high-grade tumors scanned at each center compared to those across the whole cohort (*P*=0.16). There was no significant difference between whole-tumor median parameters obtained at 1.5-tesla (T) and 3 T or between those obtained from scans acquired with the gradient echo echoplanar imaging and sPRESTO sequences, respectively (*P*>0.05 for all). Table 3 shows differences in whole-tumor median parameters between children who received a pre-bolus and those who did not.

**Table 3:** Results of Kruskal-Wallis tests comparing dynamic susceptibility-contrast MRI parameters between children who received any type of contrast agent pre-bolust versus those who did not receive a contrast agent pre-bolus

Pre-bolus contrast agent	Mean±SD		
	Uncorrected median rCBV	Corrected median rCBV	Median K_2_
Y	1.49±5.48	2.20±1.78	0.016±0.033
N	0.49±2.50	2.04±1.21	0.005±0.039
*P*-value^a^	**0.015**	0.978	**0.013**

*K*_*2*_ leakage parameter, *N* no, *rCBV* relative cerebral blood volume, *SD* standard deviation, *Y* yes

*n*=76 because injection protocol information was not available for 9 children

^a^*P* value <0.05 is significant (bold)

We found a significant difference between tumor volumes in the low- and high-grade tumor groups (mean±SD = 22.1±23.7 cm^3^ and 33.5±28.1 cm^3^; *P*=0.047). Median rCBV_uncorr_ was significantly higher in the high-grade tumor group compared to the low-grade tumor group (mean±SD = 2.37±2.61 vs. –0.14±5.55; *P*=0.008). Median rCBV_corr_ was higher in high-grade versus low-grade tumors (mean±SD = 2.54±1.63 vs. 1.68±1.36; *P*=0.01).

**Fig. 2:**
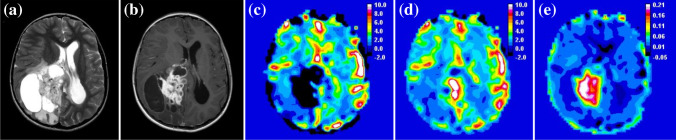
Image and maps from a low-grade tumor in an 11-year-5-month-old girl with a pilocytic astrocytoma (classified as grade I) scanned at center 4 on a 1.5-T Achieva (Philips, Best, the Netherlands). **a** Pre-contrast axial T2-weighted MR image (turbo spin-echo sequence, repetition time/echo time [TR/TE] = 6,070/100 ms, number of signals averaged [NSA] = 3, acquired with 4-mm slice thickness and 10% slice gap, reformatted to the dynamic susceptibility-contrast acquisition of 3.5-mm slice thickness, no gap and 3.4×3.4-mm in-plane resolution) on which the tumor region of interest is defined. **b** Post-contrast axial T1-weighted MR image (spin-echo sequence, TR/TE=676/12 ms, NSA=2, acquired with 4-mm slice thickness, 10% gap, reformatted as in (**a**) shows high signal in the tumor. **c–e** Uncorrected (**c**) and leakage-corrected (**d**) relative cerebral blood volume (rCBV) maps and K_2_ map (**e**) acquired in axial plane. Dynamic susceptibility-contrast data were acquired with an sPRESTO sequence (TR/TE=17/25 ms, flip angle 7^o^, with 30 slices at 3.4×3.4×3.5-mm resolution). The uncorrected rCBV map shows a black hole indicating negative values in the tumor. After correction, rCBV is shown to increase. The K_2_ map shows very high values within the tumor compared to surrounding normal tissue. Uncorrected median rCBV was negative prior to leakage correction (−9.83 vs. 2.97), K_2_ was 0.168. *sPRESTO* sensitivity-encoded Philips principles of echo-shifting with a train of observations

**Fig. 3:**
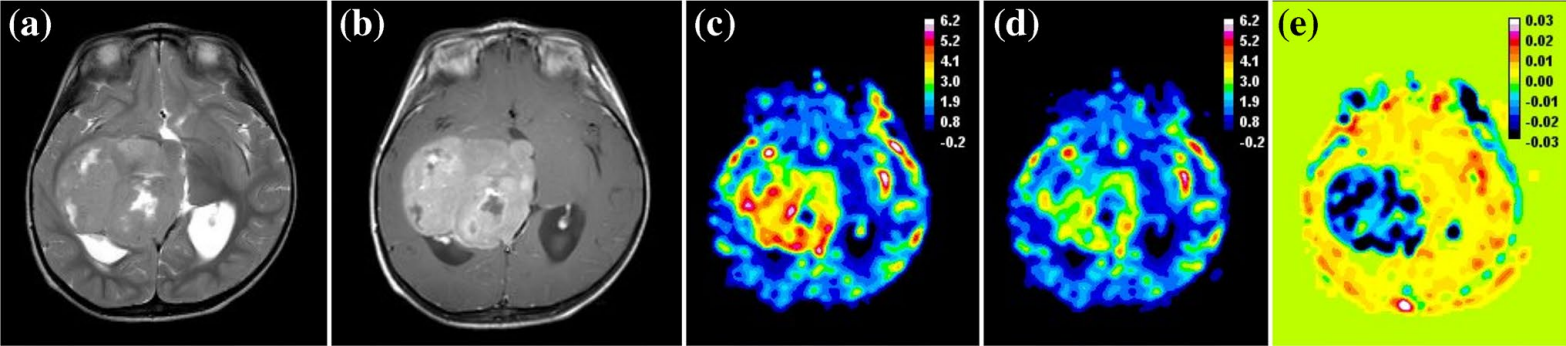
Images and maps from a high-grade tumor in a 2-year-old boy with a glioblastoma (classified as grade IV) scanned at center 4 on a 3-T Achieva (Philips, Best, the Netherlands). **a** Pre-contrast axial T2-weighted MR image (turbo spin-echo sequence, repetition time/echo time [TR/TE] = 6,272/85 ms, number of signals averaged [NSA] = 1, acquired with 4-mm slice thickness and 10% gap, reformatted to the dynamic susceptibility-contrast acquisition of 3.5-mm slice thickness, no gap and 1.8×1.8-mm in-plane resolution), on which the tumor region of interest was defined. **b** Post-contrast axial T1-weighted MR image (spin-echo sequence, TR/TE=1,179/14 ms, NSA=1, acquired with 4-mm slice thickness, 10% gap, reformatted as in (**a**) shows high signal in the tumor. **c–e** Uncorrected (**c**) and leakage-corrected (**d**) relative cerebral volume (rCBV) maps and K_2_ map (**e**) acquired in the axial plane. Dynamic susceptibility-contrast data were acquired with an sPRESTO sequence (TR/TE=15.9/23.9 ms, flip angle 7^o^, with 30 slices at 1.8×1.8×3.5-mm resolution). The uncorrected rCBV map shows high values within the tumor. After correction, rCBV is shown to decrease. The K_2_ map shows negative values within the tumor compared to surrounding normal tissue. Normalized uncorrected rCBV was high both before and after leakage correction (3.66 vs. 2.68) and K_2_ was −0.013. *sPRESTO* sensitivity-encoded Philips principles of echo-shifting with a train of observations

**Fig. 4:**
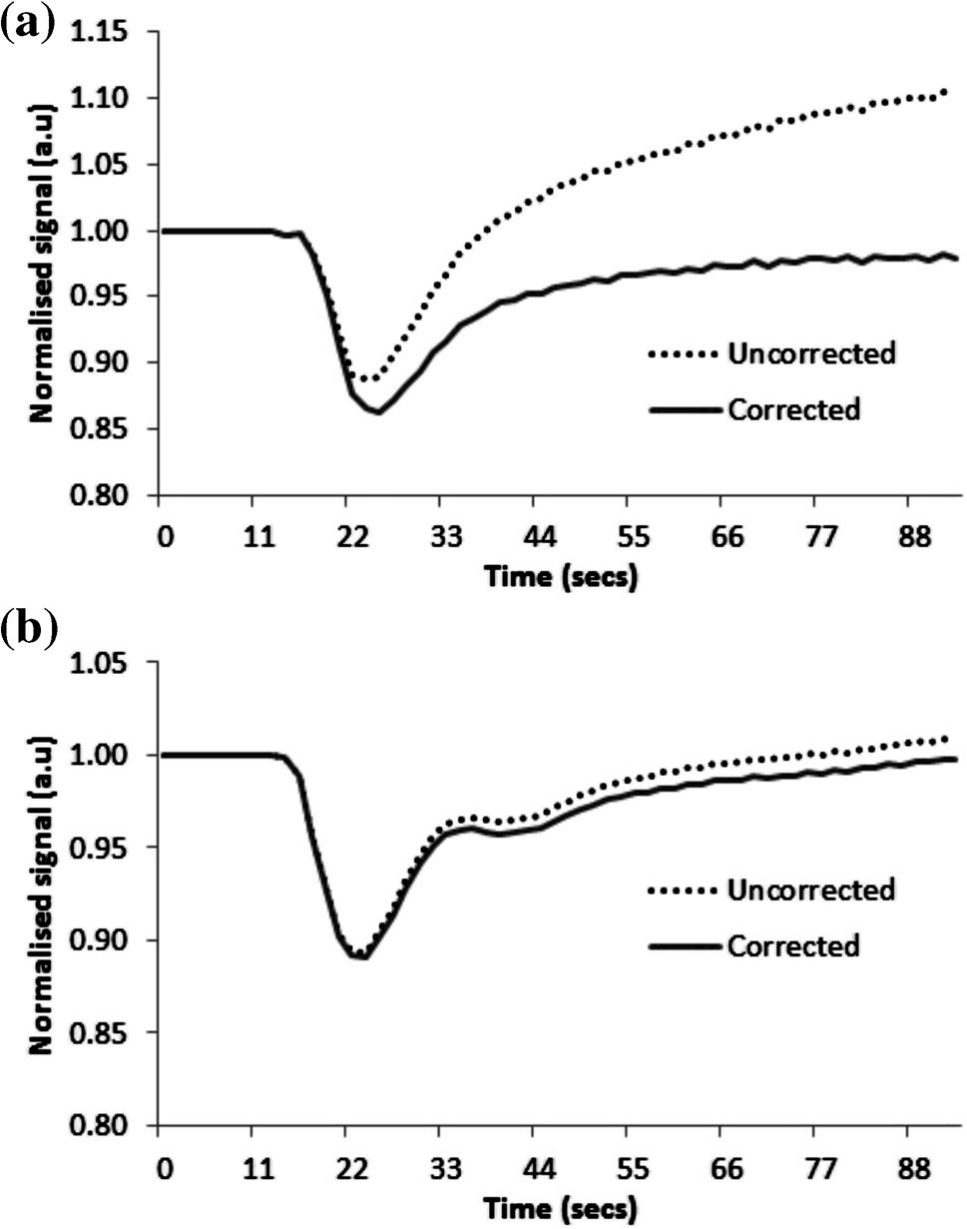
Example of signal–time curves from a low-grade tumor and a high-grade tumor, both scanned at center 2 with the same dynamic susceptibility-contrast MRI protocol. No pre-bolus contrast agent was given in either case. The uncorrected and leakage-corrected signal–time curves are shown for both tumors. **a** The low-grade tumor is a grade I pilocytic astrocytoma in a 2-year-10-month-old boy. Uncorrected and corrected median relative cerebral blood volume (rCBV) for the low-grade tumor are −0.82 and 1.08, respectively. K_2_ is large and positive at 0.022. **b** The high-grade tumor is a grade IV medulloblastoma in a 5-year-5-month-old boy. Uncorrected and corrected median rCBV for the high-grade tumor are 1.30 and 1.23, respectively. K_2_ is low at 0.001

**Fig. 5:**
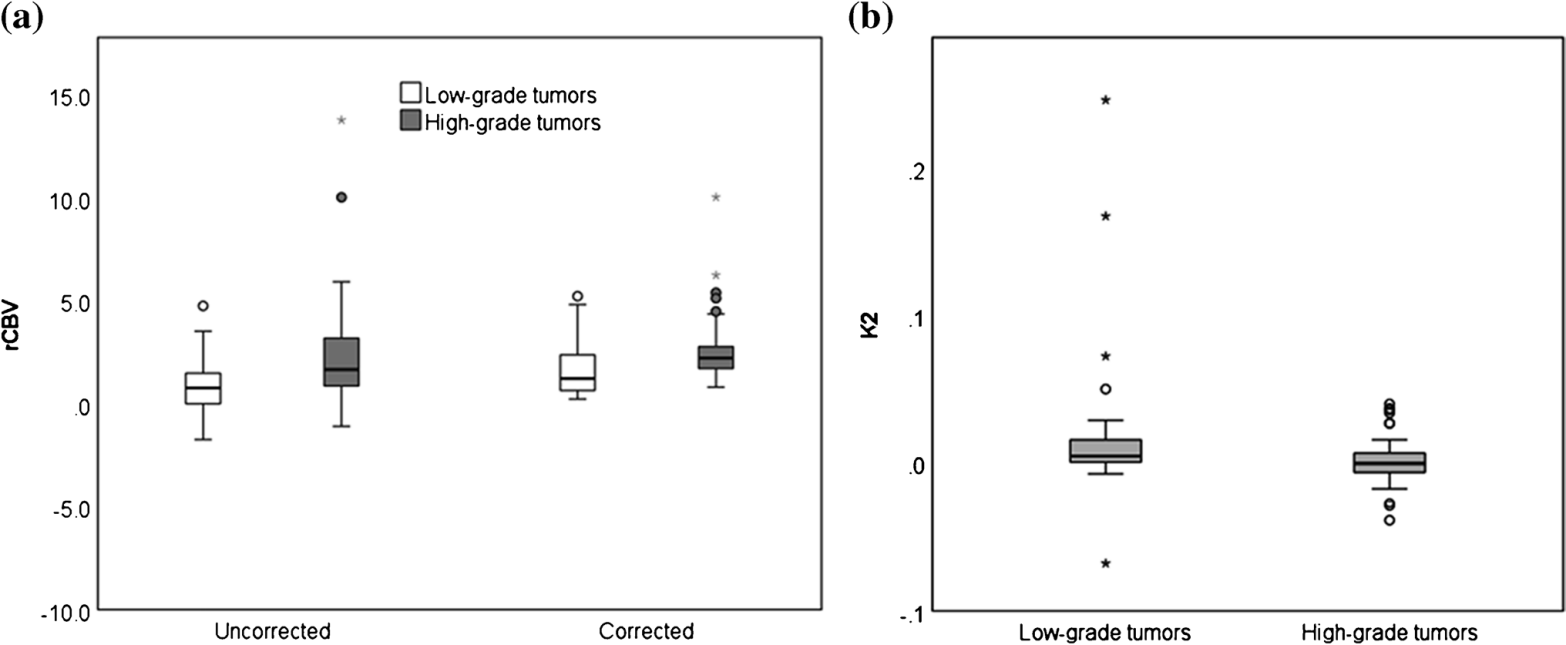
Boxplots show parameter distributions across the high-grade and low-grade tumor groups, respectively. **a, b** Median uncorrected relative cerebral blood volume (rCBV_uncorr_) and corrected relative cerebral blood volume (rCBV_corr_) (**a**) and median K_2_ (**b**). The bold lines show the mean parameter, whiskers show the interquartile range. The *P*-values from the Kruskal-Wallis tests for significant differences in parameters between the high-grade and low-grade tumor groups were significant at 0.008, 0.010 and 0.014 for rCBV_uncorr_, rCBV_corr_ and K_2_, respectively

Ten of 40 low-grade tumors had negative uncorrected rCBV, a consequence of low rCBV with high contrast agent leakage as seen on post-contrast T1-weighted MR images (Fig. 2). After leakage correction, rCBV increased in 32 of 40 low-grade tumors, including the 10 tumors where rCBV had been negative. Forty-one of 45 high-grade tumors had positive rCBV prior to leakage correction. In 19 high-grade tumors, rCBV decreased indicative of T2* effects (Fig. 3). Figure 4 shows example uncorrected and corrected signal–time curves for a low- and a high-grade tumor case, respectively. In the low-grade tumor group, a Wilcoxon signed-rank test showed that median whole-tumor rCBV increased significantly from a mean±SD of −0.14±5.55 to 1.68±1.36 following leakage correction (*P*<0.001); in the high-grade tumor group, median rCBV did not change significantly (mean±SD = 2.37±2.61 vs. 2.54±1.63; *P*=0.307; Fig. 5). Increases in median rCBV averaged across the low- and high-grade groups were 1.81 and 0.17, respectively (*P*=0.060). K_2_ was positive in 30 of 40 low-grade tumors, indicating that T1 effects dominated in this group; there was a roughly equal split between positive and negative K_2_ in the high-grade tumor group. There was a significant positive correlation between median K_2_ and change in rCBV following leakage correction (r=0.931, *P*<0.001). Low-grade tumors had significantly higher median K_2_ than high-grade tumors (mean±SD = 0.017±0.049 vs. 0.002±0.017; *P*=0.014, Fig. 5).

**Fig. 6:**
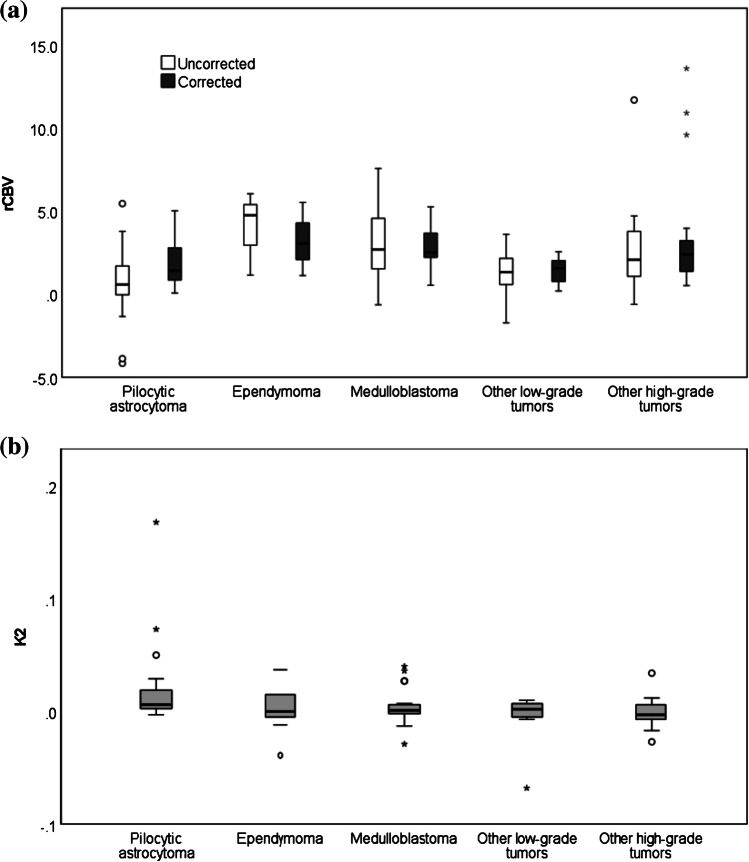
Boxplots show distribution of (**a**) median uncorrected relative cerebral blood volume (rCBV_uncorr_) and corrected relative cerebral blood volume (rCBV_corr_) and (**b**) median K_2_ across three common tumor types, with other tumors grouped as “other high-grade tumors” or “other low-grade tumors.” The *P*-values from Kruskal-Wallis tests were significant at 0.001, 0.006 and 0.035, respectively

A Kruskal-Wallis test comparing median dynamic susceptibility-contrast MRI parameters across our five tumor type groupings found that median rCBV_uncorr_ was significantly different among tumor types (*P*=0.013; Fig. 6). Post hoc testing showed that this was driven by significant differences between rCBV_uncorr_ in pilocytic astrocytomas and medulloblastomas (*P*=0.012) and between pilocytics and other horizontal gene transfer tumors (*P*<0.001). High-grade tumors, including ependymomas and glioblastomas, had the highest values. Pilocytic astrocytomas and other low-grade tumors had the lowest mean values. This significance was lost when leakage correction was applied (*P*=0.124), although rCBV_corr_ was statistically significantly different between pilocytic astrocytomas and the “other high-grade tumors” group (*P*=0.026). Median K_2_ differed significantly among tumor types (*P*=0.035). K_2_ values were highest in pilocytic astrocytomas and again were significantly different from those in the “other high-grade tumors” group (*P*=0.021), which had the lowest mean value.

**Fig. 7:**
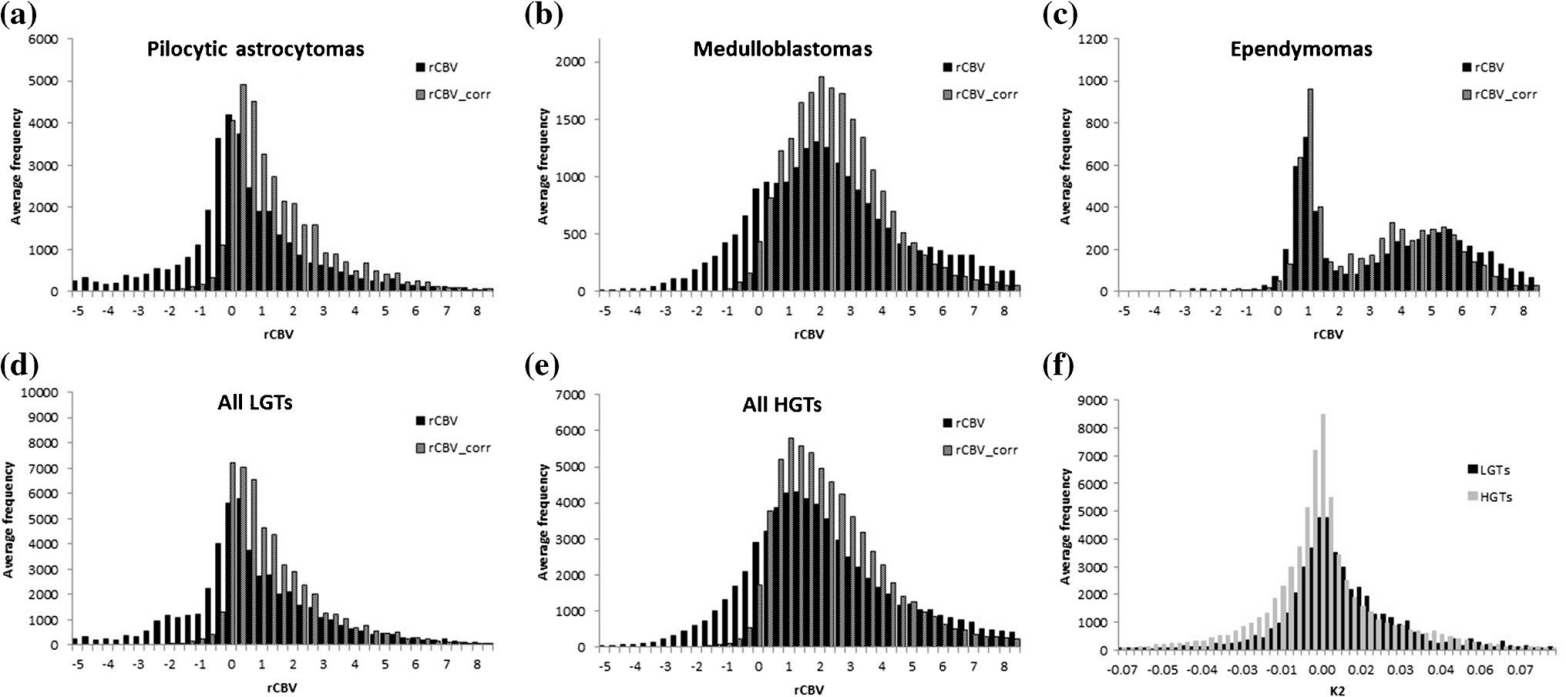
Mean histograms show the distribution of uncorrected and corrected relative cerebral blood volume (rCBV) averaged over (**a**) all pilocytic astrocytomas (*n*=27), (**b**) all medulloblastomas (*n*=23), (**c**) all ependymomas (*n*=9), (**d**) all other low-grade tumors (LGTs) (*n*=10), (**e**) all other high-grade tumors (HGTs) (*n*=7) and (**f**) the distribution of the leakage parameter (K_2_) averaged over the low- and high-grade tumor groups, respectively

**Fig. 8:**
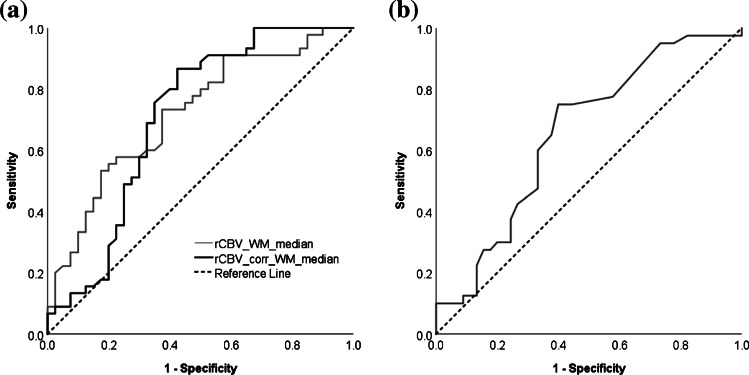
Receiver operating characteristic curves for dynamic susceptibility-contrast MRI parameters to compare performance in discriminating between high- and low-grade tumors. **a, b** Receiver operating characteristic curves for median uncorrected relative cerebral blood volume (rCBV_uncorr_) and corrected relative cerebral blood volume (rCBV_corr_) (**a**) and median K_2_ (**b**). The area under the receiver operating characteristic curve values for the parameters were 0.719, 0.707 and 0.656, respectively. *WM* white matter

Average histograms show whole-tumor uncorrected and corrected rCBV for the three most common tumor types in the study, as well as across the low- and high-grade tumor groups, respectively (Fig. 7). Table 4 shows descriptive parameters, including skew, kurtosis and key percentile values for uncorrected and corrected rCBV along with the significance of differences between the low- and high-grade tumor groups. Pineoblastomas and glioblastomas demonstrated high rCBV (corrected and uncorrected). The lowest rCBV_uncorr_ values were found in pilocytic astrocytomas, which also demonstrated the largest increase following leakage correction (from a mean of 0.13±2.23 to 1.53±1.24). Sensitivity, specificity, negative and positive predictive values of uncorrected and corrected rCBV and K_2_ are summarized in Table 5 for median cut-off values. Receiver operating characteristic curves for rCBV_uncorr_ and rCBV_corr_ and for K_2_ are shown in Fig. 8. Area under the receiver operating characteristic curve values were 0.719, 0.707 and 0.656, respectively. An rCBV_corr_ of less than 0.785 had 100% sensitivity for identifying low-grade tumors. In addition, two of the low-grade tumors with the highest rCBV_corr_ (4.09 and 2.46) were oligodendrogliomas (grade II). At the time of analysis, the child with the higher rCBV_corr_ had died while the other was still alive.

**Table 4:** Results of the Kruskal-Wallis test comparing parameters describing the distribution of uncorrected and corrected relative cerebral blood volume (rCBV) obtained from whole-tumor regions of interest between the low- and high-grade tumor groups

Parameters^a^	Tests between low- and high-grade tumor groups					
	Uncorrected rCBV			Corrected rCBV		
	Low-grade (mean±SD)	High-grade (mean±SD)	*P*-value^b^	Low-grade (mean±SD)	High-grade (mean±SD)	*P*-value^b^
Skew	0.94±1.05	0.89±0.72	0.725	1.25±1.11	1.01±0.80	0.573
Kurtosis	3.34±3.80	3.04±2.99	0.809	4.42±6.11	2.98±2.99	0.403
10th percentile	−2.08±6.82	0.36±2.05	**0.000**	−0.20±2.34	−0.86±1.09	0.154
25th percentile	−1.19±6.21	1.33±2.22	**0.000**	0.43±2.61	−0.37±0.98	0.108
75th percentile	1.18±4.76	3.51±3.23	**0.002**	1.60±3.19	0.66±1.18	**0.011**
90th percentile	2.53±4.19	4.75±3.99	**0.008**	2.13±3.39	1.11±1.40	**0.012**
Minimum	−5.19±9.67	−3.00±3.74	0.117	−0.84±2.09	−0.82±2.41	0.285
Maximum	8.43±6.19	12.00±9.02	**0.035**	8.18±6.61	9.86±8.51	0.150

*rCBV* relative cerebral blood volume, *SD* standard deviation

^a^Parameters include the skew and kurtosis in rCBV across the whole tumor, along with the values of percentiles obtained, for example, the 10th percentile value is the value below which 10% of all values in the whole-tumor region of interest lie

^b^*P*-value <0.05 is significant (bold)

**Table 5:** Mean parameters for the low- and high-grade tumor groups, respectively, along with 95% confidence intervals; cut-off values for differentiating between low- and high-grade tumors are shown alongside the specificity, sensitivity, positive and negative predictive values of each parameter when this cut-off is employed

Whole-tumor median of parameter	Grade	Mean	95% confidence interval for mean	Cut-off value	Sensitivity (%)	Specificity (%)	Positive predictive value (%)	Negative predictive value (%)
Uncorrected rCBV	Low-grade	−0.14	−1.91–1.64	1.60	53.3	82.5	77.4	61.1
	High-grade	2.37	1.58–3.15					
Corrected rCBV	Low-grade	1.68	1.24–2.11	1.70	75.6	65.0	70.8	70.3
	High-grade	2.54	2.05–3.03					
K_2_	Low-grade	0.017	0.002–0.033	0.001	60.0	75.0	73.0	62.5
	High-grade	0.002	−0.003–0.007					

*K*_*2*_ leakage parameter, *rCBV* relative cerebral blood volume

**Fig. 9:**
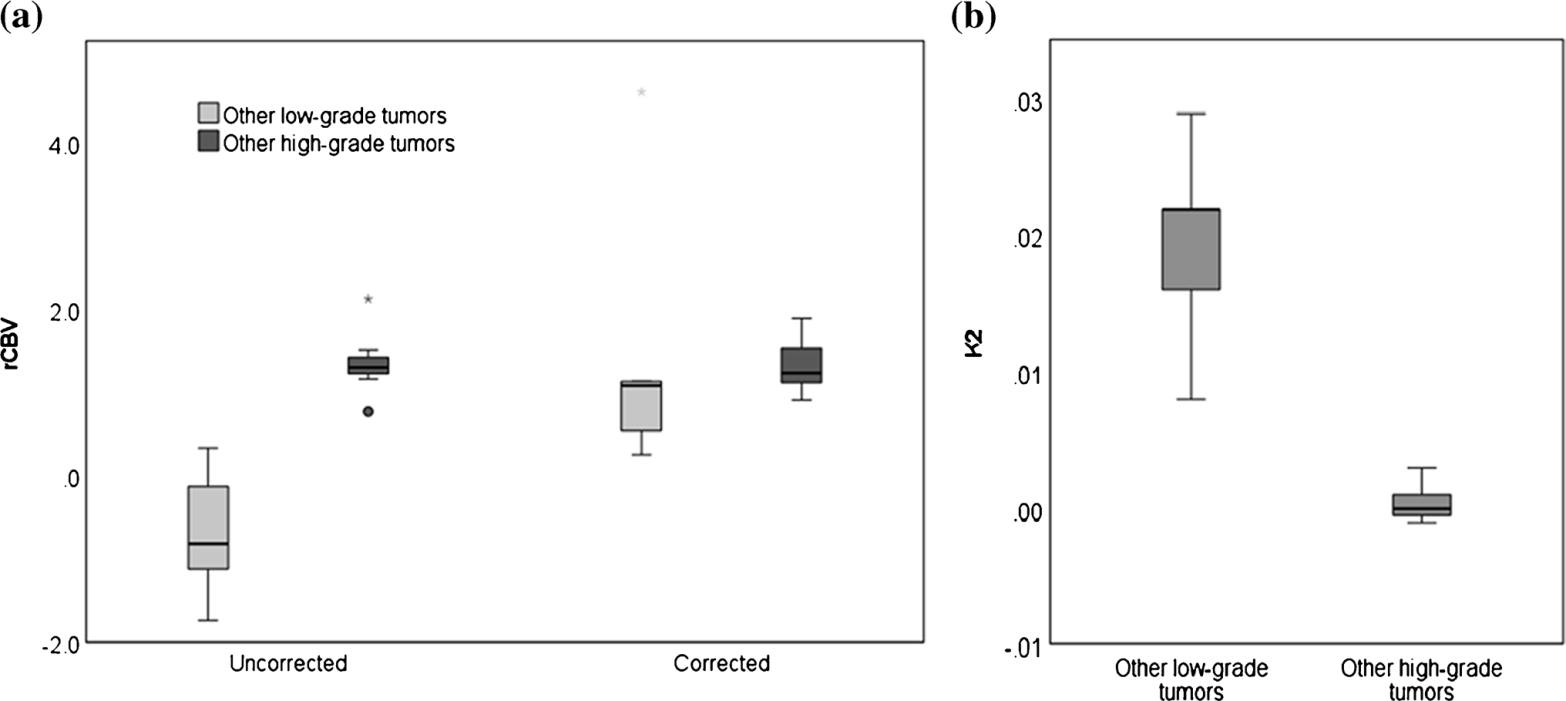
Boxplots show parameter distributions across the high-grade and low-grade tumor groups, respectively, for center 2. At center 2, children routinely did not receive a pre-bolus of contrast agent. **a** Median uncorrected relative cerebral blood volume (rCBV_uncorr_) and corrected relative cerebral blood volume (rCBV_corr_). **b** Median K_2_. The bold lines show the mean parameter value, whiskers show the interquartile range. *P*-values from Kruskal-Wallis tests for significant differences between the high- and low-grade tumor groups were <0.001, 0.782 and <0.001 for rCBV_uncorr_, rCBV_corr_ and K_2_, respectively

No significant differences were seen between median rCBV_uncorr_, rCBV_corr_ and K_2_ values measured in children scanned at different centers (*P*>0.05 in all cases). At center 2, where no pre-bolus was given, rCBV_uncorr_ and K_2_ were significantly different between the low- and high-grade tumor groups, with no overlap of values (*P*<0.005 for both rCBV_uncorr_ and K_2_; Fig. 9). At this center, K_2_ was always greater than 0.005 in the low-grade group, with high-grade tumors consistently having values below this. Corrected relative cerebral blood volume was not found to be significantly different between the groups. Using a cut-off value of 0.70, the sensitivity and specificity of rCBV_uncorr_ in children scanned at center 2 were both 100%; for rCBV_corr_, they fell to 71% and 80%, respectively, with a cut-off of 1.15; for K_2_, 100% sensitivity and specificity were achieved using a cut-off of 0.005.

## Discussion

We found significant differences in uncorrected and leakage-corrected rCBV when comparing low- and high-grade pediatric brain tumors scanned prior to treatment at multiple centers. This finding is despite large differences in the dynamic susceptibility-contrast MRI protocols employed across centers. While other single-center pediatric studies have shown that rCBV can discriminate between high- and low-grade brain tumors [1, 2, 15, 24, 25], this has not been established in a large multi-center study, and while leakage correction was often used, the results were generally not presented. By analyzing data acquired at multiple centers, we included many children with a variety of tumor types. In particular, pilocytic astrocytomas tended to have low perfusion despite being contrast-enhancing, and an important message of this study is that leakage correction in this tumor group is particularly important if an erroneous rCBV is to be avoided. Overall, these results suggest that, despite differences in dynamic susceptibility-contrast MRI protocols, data can be used to aid clinicians in classifying tumors as low- or high-grade.

Uncorrected rCBV was the most significant parameter for discriminating between high- and low-grade tumors. Low-grade tumors, particularly pilocytic astrocytomas, often had negative rCBV. On applying leakage correction, rCBV became positive, reducing the significance of differences between values in the low- and high-grade tumor groups. Other studies [1, 2] found significantly higher rCBV in high- compared to low-grade tumors. Overlap between values in the two groups in both of those studies as well as ours is extensive, suggesting that dynamic susceptibility-contrast MRI results alone should not be used for tumor grading but should be viewed in conjunction with other MR imaging. Diffusion-weighted imaging in addition to dynamic susceptibility-contrast MRI was found to have high predictive diagnostic accuracy when grading pediatric brain tumors [15]. In a clinical setting, dynamic susceptibility-contrast MRI should be part of a diagnostic pathway that is refined in a stepwise manner as more information becomes available, starting with clinical history and examination findings, being refined by conventional imaging and then advanced MRI. In this way, perfusion might provide reassurance in the putative diagnosis, or challenge it. In general, the greater the perfusion, the greater the suspicion would be that the tumor is of a high grade.

In Ho et al. [2], pilocytic astrocytomas had the lowest maximum rCBV. High-grade atypical teratoid rhabdoid tumors, medulloblastomas and ependymomas had the highest maximum rCBV. We found that only glioblastomas and pineoblastomas had higher rCBV than ependymomas, with medulloblastomas having the next highest rCBV. A previous study showed that of all gliomas included, glioblastomas had the highest maximum white-matter-normalized rCBV, 7.32 [26]. Some tumor groups in our study were limited in number and so results should be treated with caution. Medulloblastomas had a large range of rCBV values, possibly because of differences in molecular subgroups [27]. Ho et al. [2] also presented average histograms of rCBV for different tumor types, showing that pilocytic astrocytomas had a higher proportion of low rCBV values than high-grade tumors including ependymomas and medulloblastomas. Similarly, we found differences between rCBV histogram centiles. As in Ho’s study, skew and kurtosis did not differ significantly between the low- and high-grade groups in our study, suggesting that while histograms from different tumor types might differ in shape, when taken over a larger population, these differences are not significant.

Another study [24] did not employ leakage correction but classified dynamic susceptibility-contrast MRI signal–time courses as having no leakage, T1- or T2*-dominant leakage depending on whether they returned to baseline, continued above baseline or failed to return to baseline, respectively. Sensitivity tests found that a T1-dominant leakage pattern predicted lateral gene transfer in 66% of cases, rising to 91% in pilocytic astrocytomas; a T2*-dominant or baseline pattern predicted horizontal gene transfer in 100% of cases. We found median rCBV_corr_ had the highest sensitivity (76%) and specificity (65%) for detecting high-grade tumors using a cut-off of 1.70. A threshold of 1.60 for rCBV_uncorr_ resulted in reduced sensitivity (53%) while specificity was improved (83%). These cut-off values lie above and below the 1.38 for maximum rCBV found in Ho et al. [2] and 1.07 for rCBV resulting in 100% sensitivity found by Dallery et al. [1], but they are close to the 1.75 cut-off presented by Law et al. [28] when grading adult gliomas. The low sensitivity and specificity found in our study again emphasize the importance of not using these as single tests but rather as adding information to other clinical and imaging characteristics to achieve the most likely noninvasive diagnosis, with rCBV values well above or below the cut-off having more influence. While cases that have an rCBV_corr_ close to the cut-off cannot be confidently assigned as low- or high-grade, those with a value below 0.785 are highly likely to be a low-grade tumor. Similarly, tumors with an rCBV_corr_ that is much higher than the cut-off might have an aggressive phenotype even if low-grade, as seen in the two oligodendrogliomas, known to be the more aggressive of pediatric low-grade tumors. It could be that dynamic susceptibility-contrast MRI parameters give prognostic information, as has been shown in other pediatric studies [4].

Only one pediatric study has presented K_2_ results. Provenzale et al. [25] found that K_2_ was significantly higher in high-grade than in low-grade tumors. This contradicts our results, although the leakage-correction model used [10] differs from ours [9] in not including correction for T2*-dominant effects. In Liu et al. [9], T1-dominant tumors had higher, positive K_2_ values whereas T2*-dominant tumors had lower, often negative, K_2_ values. In agreement with Dallery et al. [1], pilocytic astrocytomas demonstrated significant T1 effects, suggesting that K_2_ should be raised in these tumors. K_2_ provides a measure of the amount of leakage correction that has been applied and so will be reduced by the administration of a pre-bolus of contrast agent. It is known to depend on sequence parameters (TR, TE), pre-contrast T1 value, blood volume and permeability–surface area product. Studies have shown that K_2_ correlates well with K_trans_ obtained from dynamic contrast-enhanced MRI [29], which represents a combination of permeability–surface area product and blood flow [30].

K_2_ and rCBV_uncorr_ had lower sensitivity and specificity than rCBV_corr_, likely caused by differences in injection protocols and the need to correct for negative rCBV_uncorr_ values. Leakage correction is therefore essential to improve the accuracy of rCBV values and to account for differences in injection protocols across a pooled dataset such as this, improving the differentiation of the low- and high-grade groups. Uncorrected relative cerebral blood volume and K_2_ should be treated with caution unless injection protocols are consistent across the patient population. While we have presented thresholds that maximize the sensitivity and specificity at identifying high-grade tumors across this whole dataset, thresholds vary with the protocol used and so the optimal threshold should be established on a site-by-site basis.

Previous studies recommended administering a pre-bolus of contrast agent to minimize T1 effects in enhancing brain tumors that result in underestimation of uncorrected rCBV [6, 7]. While a pre-load of contrast agent reduces the effects of leakage, it does not eliminate them. This is particularly the case in the pediatric population, where administration of a single dose of gadolinium is recommended, being split between the pre-bolus and the main bolus. Consequently, if the size of the pre-bolus is increased, then leakage effects are more successfully suppressed but the size of the main bolus is reduced, leading to a reduced signal drop to noise ratio of the time-course. Use of leakage correction reduces the variability that results from the use of different pre-bolus volumes and is particularly useful if rCBV is to be compared across multiple protocols employing different extents of leakage suppression by use of a pre-bolus, as in our study. K_2_ and rCBV_uncorr_ depend on the size of pre-bolus given, whereas a leakage-corrected rCBV should be more robust, and in pooled data with multiple injection protocols, leakage correction is essential to provide comparable data.

One center in our study consistently did not employ a pre-bolus of contrast agent. Leakage-uncorrected rCBV in the high- and low-grade tumor groups from this center had the best separation and highest significance using a cut-off of 0.70. K_2_ was always greater than 0.005 in low-grade tumors, with high-grade tumors consistently having values below this, suggesting that K_2_ can differentiate between low- and high-grade tumors and might hold valuable information if comparing across a dataset with consistent injection protocols. These results, while interesting, should be viewed with caution — only 12 children were scanned at this center, although there was a good split between low-grade (*n*=5) and high-grade (*n*=7) tumors. We also suggest that a pre-bolus of contrast agent is not necessary if leakage correction is applied and that administering a pre-bolus affects K_2_ values by compensating for leakage correction. It was recently reported [31] that a pre-bolus might not be necessary in adult brain tumors and that a low-flip-angle protocol with leakage correction might be preferable [32]. In our study, children who received a pre-bolus of contrast agent had increased uncorrected rCBV (suggesting a reduction in T1 leakage effects) and reduced K_2_ (indicating reduced need for leakage correction). Leakage correction reduced differences in rCBV from injection protocol. It should be noted that we did not test for all differences in injection protocols, comparing only between those who received a pre-bolus and those who did not.

While too many differences exist between the dynamic susceptibility-contrast MRI protocols in this study to draw any conclusions regarding protocol optimization, certain factors (field strength and pulse sequence) did not result in significant differences between parameters obtained across the dataset. This suggests that differences in median parameters between high and low tumor grading are greater than those introduced by the variation in scan protocols.

Data in this study were acquired at multiple centers with variable protocols, creating challenges for data analysis. Thirty-two datasets were excluded because of technical issues — data corruption, incomplete data and poor quality. While the sPRESTO and gradient echo echoplanar imaging sequences produced comparable cerebral blood volumes in simulations and animal studies [18], poor temporal stability has been observed with the sPRESTO sequence [33]. Signal-to-noise ratio was variable between protocols, reduced by use of a pre-bolus and low flip angle, while the use of 3 T and no pre-bolus boosted signal-to-noise ratio. Trade-offs were made between spatial resolution and whole-head coverage versus temporal resolution and signal-to-noise ratio. All centers administered a standard single dose of contrast agent in line with current recommendations [17]. Reproducibility of parameters from regions of interest defined by two users suggests that region definition can be undertaken by multiple users across centers. We defined regions of interest encompassing the whole tumor to investigate differences in whole-tumor median parameters as well as the distributions of parameters across the tumor. Other studies have measured rCBV in hot spots, showing significant differences in maximal perfusion in the tumor. Choosing a hot spot is subject to location, being affected by both protocol and analysis method, and has a risk of being unduly affected by artifacts [5]; therefore, we expect a whole-tumor method to be more robust in a multicenter study. Finally, there were three versions of the World Health Organization guidance on classifying central nervous system tumors over the long accrual period in this study [19–21]. Tumors were classified according to the guidance available at the time. Tumor gradings were not affected by any changes.

In recent years, there have been concerns about the use of gadolinium contrast agents. People with poor renal function have been shown to be at risk of developing nephrogenic systemic fibrosis following gadolinium exposure [34], while recent studies have shown increased signal caused by T1 shortening on MRI scans from contrast agent deposition in areas of the brain including the dentate nucleus and globus pallidus [35] following earlier exposure to gadolinium. Children are at of low risk of nephrogenic systemic fibrosis [36]; however, there are concerns about the long-term effects of gadolinium deposition in children’s brains, particularly in those undergoing repeated MR examinations with contrast agents [37]. Guidance mandates use of macrocylic rather than linear agents to minimize risks, the use of single dose titrated by weight, and risk-versus-benefit analysis before prescribing contrast agent, with consideration given to non-contrast methods [38]. In patient groups such as children with brain tumors, it is still recommended that a single-dose contrast agent be administered during MRI scans at diagnosis and follow-up for acquisition of post-contrast conventional MRI [17] and, while this remains the case, acquiring dynamic susceptibility-contrast MRI after the contrast injection has no added risks compared to the routine imaging. Indeed, it provides an efficient use of resources. A power injector is recommended for reproducible administration of contrast agent during dynamic susceptibility-contrast MRI [17, 32]. This requires venous access via a cannula, which is invasive and can be tricky, particularly in children [39]. The majority of our pediatric brain tumor patients have a cannula in situ at the time of their staging scan or because they are undergoing an MRI under general anesthetic.

Arterial spin-labeling measures perfusion without the need for a contrast agent. It has been shown to agree with dynamic susceptibility-contrast MRI measures of perfusion in children [40], with increased perfusion observed in high-grade pediatric brain tumors compared to low-grade tumors [14]. It is gaining popularity as a method, particularly in populations at risk of nephrogenic systemic fibrosis or in those undergoing repeat MRIs. However, in comparison to dynamic susceptibility-contrast MRI, it suffers from long scan times, low signal-to-noise ratio and poor spatial resolution, and leakage information, shown to be of use in this study, is not available. Arterial spin labeling is difficult in children because of age-related variations in blood flow. While there is a recommended protocol for clinical applications in adults [41], this method is difficult in children, where the optimal post-labeling delay required has been shown to vary with age [42]. Other advanced MRI methods, including MR spectroscopy and diffusion-weighted MRI, have also been shown to provide information on tumor grading in pediatric brain tumors [43].

## Conclusion

Despite difficulties in dealing with multicenter data, we have shown that rCBV values derived from dynamic susceptibility-contrast MRI data acquired at multiple centers can be used to help discriminate between high- and low-grade pediatric brain tumors. Perfusion parameters varied with tumor type but not with center. Low-grade tumors had significantly lower rCBV than high-grade tumors, requiring leakage correction to counteract T1-dominant effects. Thresholds using the median parameter of 1.60 and 1.70 for uncorrected and corrected rCBV, respectively, gave moderate sensitivity and specificity for identifying high-grade tumors. Dynamic susceptibility-contrast MRI without a pre-bolus of contrast agent gave improved sensitivity and specificity for rCBV_uncorr_ and K_2_ in a small subset of children, suggesting that a pre-bolus could be omitted in this population. Leakage-corrected dynamic susceptibility-contrast MRI in conjunction with conventional MRI and other advanced MR techniques, such as diffusion-weighted imaging and spectroscopy, might aid in early grading of pediatric brain tumors.
